# Characterization of key enzymes involved in triacylglycerol biosynthesis in mycobacteria

**DOI:** 10.1038/s41598-021-92721-y

**Published:** 2021-06-24

**Authors:** Agostina Crotta Asis, Franco Savoretti, Matías Cabruja, Hugo Gramajo, Gabriela Gago

**Affiliations:** 1 Laboratory of Physiology and Genetics of Actinomycetes, Instituto de Biología Molecular y Celular de Rosario (IBR-CONICET), Facultad de Ciencias Bioquímicas y Farmacéuticas, Universidad Nacional de Rosario, Rosario, Argentina; 2 Present Address: Stanford University, Stanford, USA

**Keywords:** Microbiology, Bacteriology

## Abstract

Phosphatidic acid phosphatase (PAP) catalyzes the dephosphorylation of phosphatidic acid (PA) yielding diacylglycerol (DAG), the lipid precursor for triacylglycerol (TAG) biosynthesis. PAP activity has a key role in the regulation of PA flux towards TAG or glycerophospholipid synthesis. In this work we have characterized two *Mycobacterium smegmatis* genes encoding for functional PAP proteins. Disruption of both genes provoked a sharp reduction in de novo TAG biosynthesis in early growth phase cultures under stress conditions. In vivo labeling experiments demonstrated that TAG biosynthesis was restored in the ∆PAP mutant when bacteria reached exponential growth phase, with a concomitant reduction of phospholipid synthesis. In addition, comparative lipidomic analysis showed that the ∆PAP strain had increased levels of odd chain fatty acids esterified into TAGs, suggesting that the absence of PAP activity triggered other rearrangements of lipid metabolism, like phospholipid recycling, in order to maintain the wild type levels of TAG. Finally, the lipid changes observed in the ∆PAP mutant led to defective biofilm formation. Understanding the interaction between TAG synthesis and the lipid composition of mycobacterial cell envelope is a key step to better understand how lipid homeostasis is regulated during *Mycobacterium tuberculosis* infection.

## Introduction

Tuberculosis (TB) is one of the top 10 causes of death worldwide and the leading cause of death from a single infectious agent (ranking above HIV/AIDS). In 2019, about 10 million people developed TB and 1.4 million died^1^. TB is caused by the bacillus *Mycobacterium tuberculosis* and can persist for decades within the granuloma in a dormant state referred to as latency. In this state, most bacilli are confined to the granuloma where a specific population of macrophages, known as foamy macrophages, are enriched in lipid droplets^2,3^. In addition, *M. tuberculosis* accumulates intracytoplasmic lipid inclusions (ILI) in its own cytoplasm that mainly contain triacylglycerol (TAG)^4^. Interestingly, TAG accumulation has been reported in several actinomycetes including the *Mycobacterium* genus^5^. ILI formation has been observed in slow- and fast-growing mycobacteria such as species from the *M. tuberculosis* complex^6–8^, *M. leprae*^9^, *M. avium*^10^, *M. marinum*^11,12^, *M. abscessus*^13^, *M. smegmatis*^14,15^ and *M. ratisbonense*^16^*.* Mycobacteria accumulate TAG in response to several growth-limiting stresses but the physiological role of this response remains unclear^17,18^.

De novo biosynthesis of TAG occurs by the sequential esterification of glycerol-3-phosphate producing phosphatidic acid (PA). PA is a key molecule for the biosynthesis of membrane glycerophospholipids through the synthesis of the common precursor CDP-diacylglycerol (CDP-DAG)^19^. However, in oleaginous bacteria PA can also be dephosphorylated by a phosphatidic acid phosphatase (PAP) to yield diacylglycerol (DAG), which is in turn acylated by the wax ester/DAG acyltransferases enzymes (WS/DGAT) to synthetize TAG^20^ (Supplementary Fig. S1). Thus, DAG formation is the first committed reaction of TAG biosynthesis, suggesting a key role of PAP activity in the regulation of PA flux towards TAG or membrane phospholipid biosynthesis. Remarkably, despite the relevance of PAP activity for the biosynthesis of TAGs, there are no studies addressing the physiological role of PAPs in mycobacteria.

Two families of PAP enzymes, Mg^2+^-dependent (PAP type 1, PAP1) and Mg^2+^-independent (PAP type 2, PAP2), have been characterized in higher eukaryotic cells and microorganisms^21^. The PAP1 family utilizes PA as a unique substrate and is localized in the soluble fraction of the cell^22^. In contrast, the PAP2 enzymes, also known as lipid phosphate phosphatases (LPPs), can use PA, lysophosphatidic acid (LPA), sphingosine- 1-phosphate and DAG pyrophosphate (DGPP) as substrates and are integral membrane proteins. Although PAP2 enzymes have been found both in eukaryotic and prokaryotic organisms, PAP1 enzymes are highly conserved in eukaryotes but absent in prokaryotes. Moreover, only a few PAP2-like enzymes have been characterized in prokaryotes^23–25^. The first PAP2 enzyme characterized in bacteria was PgpB of *E. coli*^23^. PgpB had a broad substrate spectrum like PGP, PA, LPA, DGPP and undecaprenyl pyrophosphate (C55-PP)^23,26^. Furthermore, Comba et al.^25^ characterized two PAP2 enzymes in *Streptomyces coelicolor*, Lppα and Lppβ, that showed significant PAP activity when expressed in *E. coli*. Analysis of mutant and overexpressing strains of these enzymes in *S. coelicolor* determined a direct link of these enzymes with TAG biosynthesis in oleaginous bacteria.

In this study we present the genetic and physiological characterization of two PAPs from *M. smegmatis*, demonstrating that *msmeg_0633* (PAPα) and *msmeg_0634* (PAPβ) encode PAP enzymes catalyzing the formation of DAG from PA. Furthermore, we demonstrated that the absence of PAP activity triggers changes in lipid metabolism in order to maintain TAG levels, highlighting the relevance of TAG synthesis in theses microorganisms.

## Results

### Identification of putative PAPs in *M. smegmatis*

In order to identify PAP enzymes responsible for DAG production in *M. smegmatis*, we performed a BLAST homology search^27^ over different actinomycetes genome databases using the SCO1102 amino acid sequence as query. SCO1102, named Lppα, has been previously identified and characterized as a PAP protein in *S. coelicolor*^25^. The output of this search indicated the presence of several proteins belonging to the PAP2 superfamily in mycobacteria. In particular, two putative PAPs were identified in *M. smegmatis*, MSMEG_0634 and MSMEG_0633. On the other hand, *M. tuberculosis* only revealed one PAP candidate, orthologous to MSMEG_0634. Interestingly, MSMEG_0633 protein presents a diacylglycerol kinase catalytic domain (pfam 00781) besides the PAP2 superfamily domain (pfam 01569).

**Figure 1:**
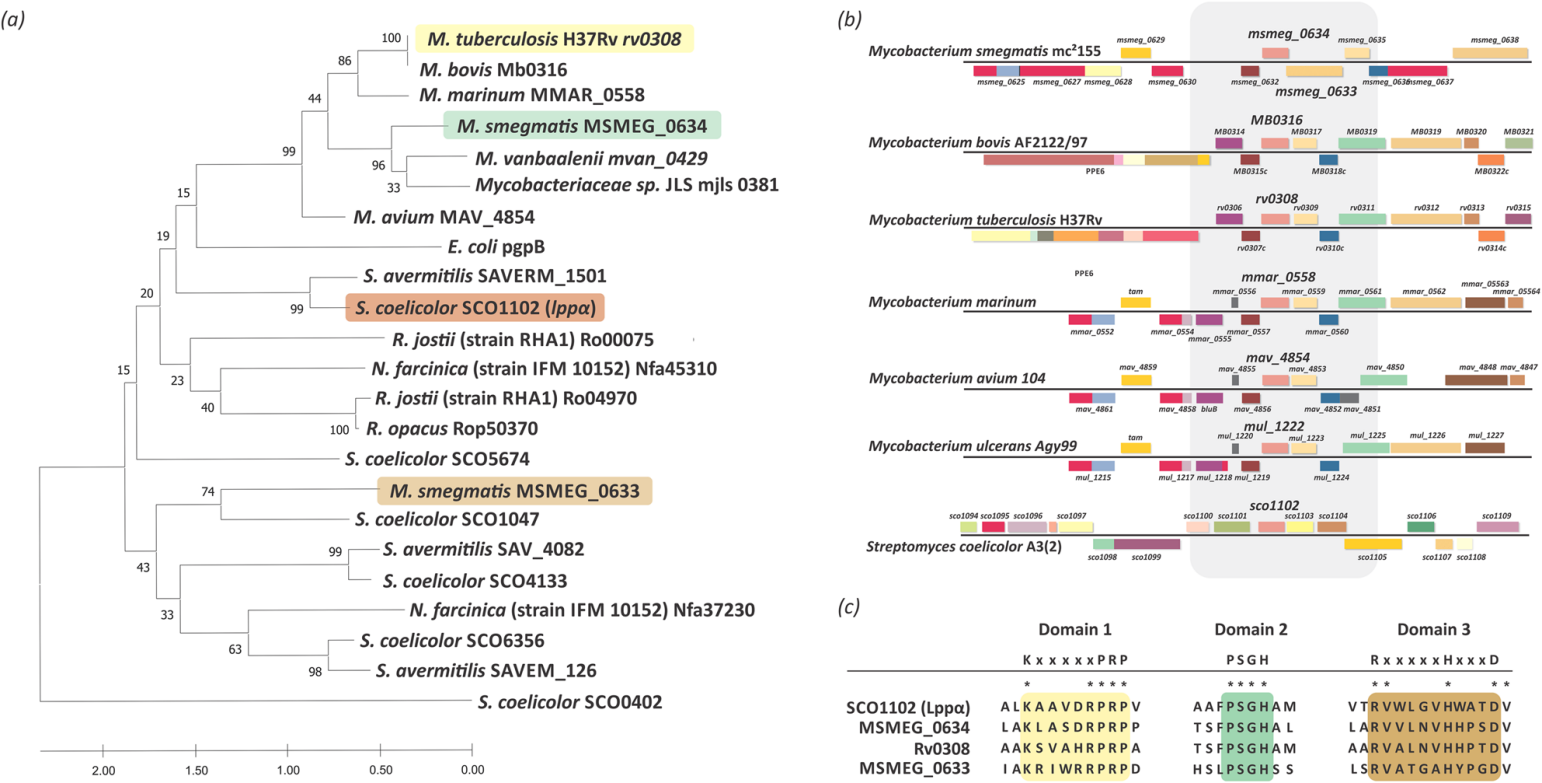
Bioinformatic analysis of PAPs. (**a**) Phylogenetic tree of PAP enzymes from different actinomycetes. The maximum likelihood phylogenetic tree was constructed using the MEGA X software. The bootstrap percentage supports (1000 replicates) are indicated in the different branches. (**b**) Comparative analysis of the genomic context of putative PAP enzymes from mycobacteria and *Streptomyces coelicolor*. (**c**) Sequence alignment of PAP2 domains. The key residues for this catalytic activity are highlighted.

Phylogenetic analysis using curated sequences of PAP2 from several actinomycetes showed that MSMEG_0634 and Rv0308, as well as other putative PAP found in other mycobacteria*,* clustered with SCO1102 previously characterized in *S. coelicolor*^25^. MSMEG_0633 clustered together with other PAP proteins from *Streptomyces* such as SCO1047 (Fig. 1a).

Using the Microbial Genome Database (MBGD) tool we analyzed the genetic environment of the coding genes for these enzymes and found that they are highly conserved in several mycobacteria such as *M. avium, M. ulcerans, M. bovis* and *M. marinum* as well as *M. smegmatis* and *M. tuberculosis*. However, this synteny was not conserved in *S. coelicolor* (Fig. 1b). Detailed analysis of the protein sequences found in *M. tuberculosis* and *M. smegmatis* indicated that they all conserved the key amino acids present in the PAP2 catalytic domain (Fig. 1c).

### Heterologous expression of *M. smegmatis *PAPs enzymes results in an increase in DAG levels in *E. coli*

**Figure 2:**
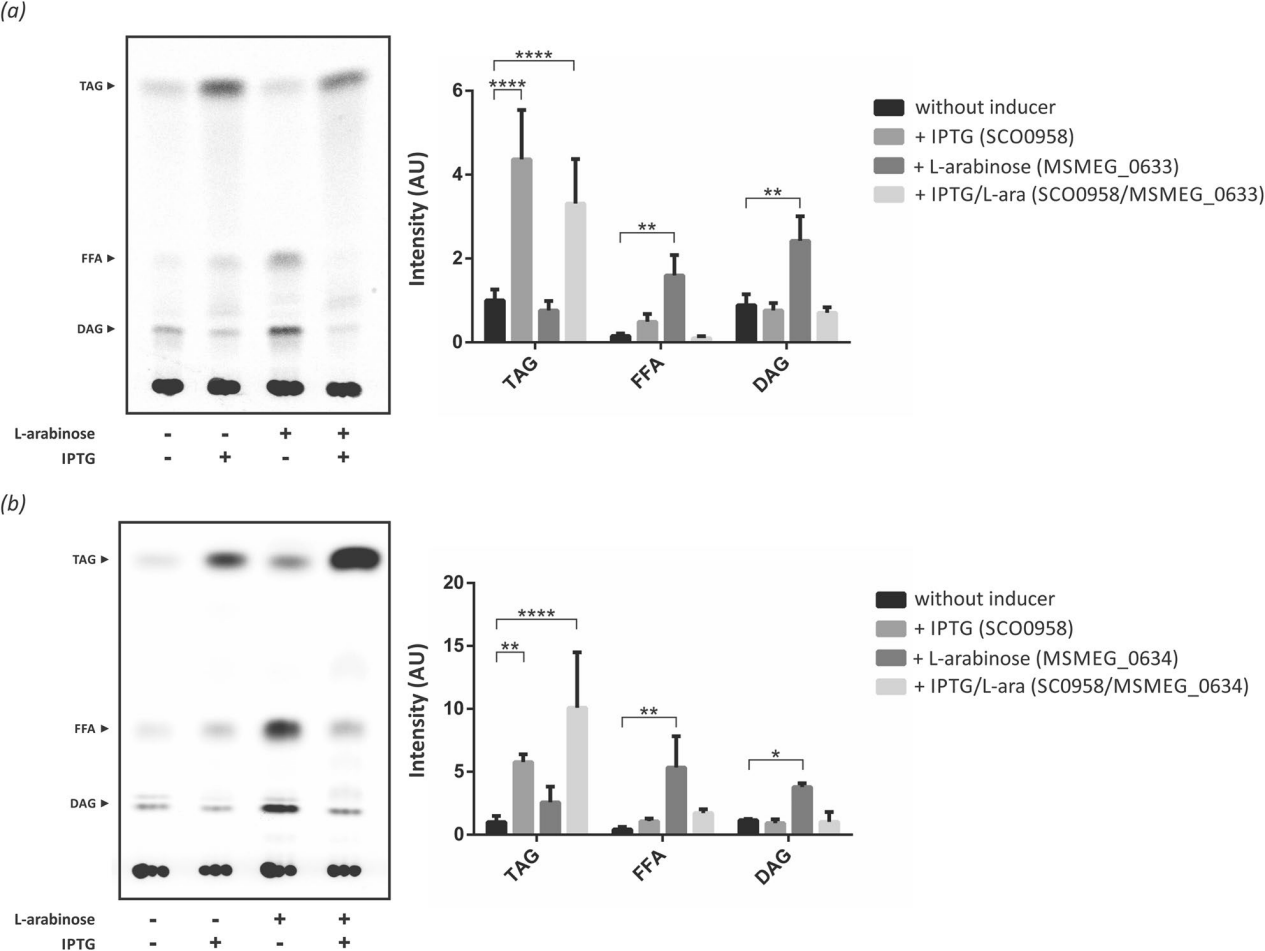
Heterologous expression of MSMEG_0633 and MSMEG_0634 proteins in *E. coli*. Total lipid extracts from [^14^C]-acetate labelled cultures of *E. coli* strains GG-03 (**a**) or GG-02 (**b**) were analyzed on silica gel TLC plates and developed in hexane: diethylether:acetic acid (70:30:1, v/v/v). Quantification of the radiolabelling intensity is shown for both TLC in arbitrary units (AU). The results are the mean values and standard deviation of three independent biological replicates. All results were normalized by OD_600nm_ and total protein concentration. TAG, triacylglycerol; FFA, free fatty acids; DAG, Diacylglycerol. (+): Inducer added. (−): Without inducer; AU, arbitrary units. Statistical significance was calculated using two-way ANOVA test followed by Bonferroni correction; ****P < 0.0001; ***P < 0.001; **P < 0.01; *P < 0.05.

To initiate the functional characterization of MSMEG_0633 and MSMEG_0634, an N-terminal His-tag version of each gene was cloned under the control of *P*_*BAD*_ promoter in the pBAD33 vector^28^. Plasmids pAC35 and pAC6 were introduced by transformation in the BL21 derivative strain MPS11, a *dgk* mutant heterologous expressing the DAG:acyltransferase (DGAT) SCO0958 from *S. coelicolor* under the control of T7 promoter^29^. Transformed cells were grown to mid-log phase and then cultivated for 16 h at 23 °C after induction with L-arabinose and/or IPTG. We analyzed the lipid profile of these recombinant strains by metabolic labeling with [^14^C]-acetate. As shown in Fig. 2a,b, we found that the expression of MSMEG_0633 and MSMEG_0634 increased the intracellular levels of DAG by 3 and fourfold, respectively compared with the same strain grown without the addition of the inducer (Fig. 2). The free fatty acid content of GG-02 and GG-03 strains expressing MSMEG_0634 and MSMEG_0633, respectively, also increased compared with the non-induced strains.

The sole expression of SCO0958 in the MPS11 strain was sufficient to promote accumulation of significant levels of TAG. However, co-expression of both SCO0958 and MSMEG_0634 enzymes in strain GG-02 increased TAG production by 12-fold (Fig. 2b), suggesting that the expression of these enzymes are sufficient to synthesize de novo TAG using precursors from the glycerophospholipid metabolism of a non-oleaginous host. Altogether, our results suggest that MSMEG_0633 and MSMEG_0634 are central enzymes for DAG generation in *M. smegmatis* and we named them as msPAPα and msPAPβ.

### Construction and analysis of single mutant strains in PAP enzymes

In order to study the physiological role of msPAPα and msPAPβ in storage lipid synthesis we generated two single knockout (KO) mutant strains in the *M. smegmatis* genes *msPAPα* and *msPAPβ*. For this, we constructed two pPR27-derived plasmids, using the upstream and downstream flanking fragments of each gene (Supplementary Fig. S2), and we called them pAC21 and pAC17, respectively. These vectors can be efficiently counterselected on sucrose at 42 °C. The correct double recombination events leading to the deletion of each PAP coding gene were confirmed by genomic PCR (Supplementary Fig. S2) and the resulting mutant strains named ∆PAPα (*msmeg_0633* KO) and ∆PAPβ (*msmeg_0634* KO)*,* respectively*.*

To study the effect of each of the PAP protein depletion at different growth stages, the two mutant strains were grown in 7H9 medium and TAG accumulation analyzed by total lipid extraction and fractionation by normal-phase TLC. Both strains showed exponential growth curves comparable with the wild type. However, ∆PAPα presented a longer *lag* phase and a delayed entry in the exponential phase (Supplementary Fig. S3). None of the single mutants showed reduction in TAG accumulation under these conditions and surprisingly, ∆PAPβ was able to accumulate even more TAG during exponential phase compared with the wild type strain.

### PAP depletion results in reduced TAG biosynthesis in early growth phase under stress conditions

**Figure 3:**
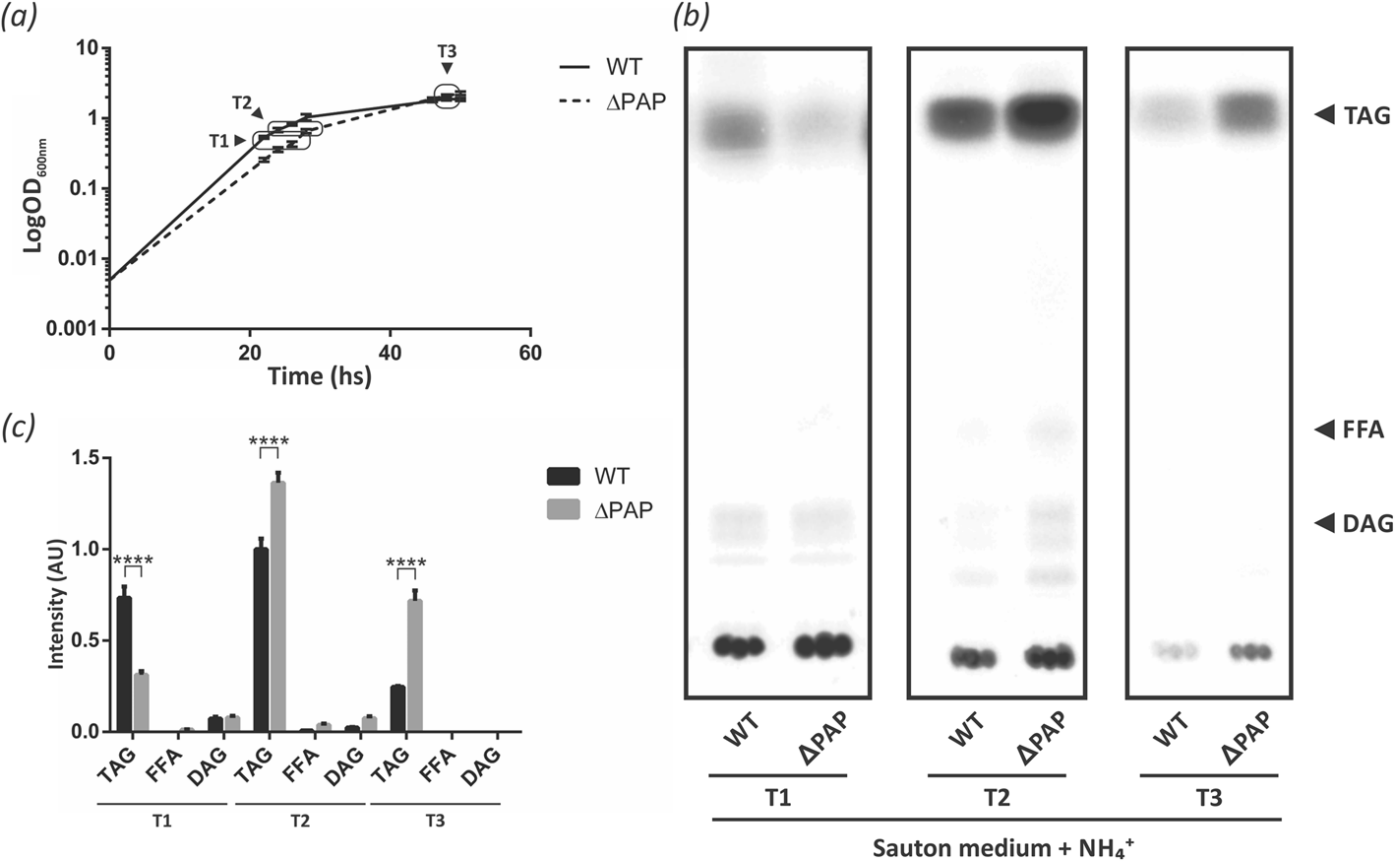
Growth curve and TAG production of *M. smegmatis* ∆PAP in low ammonium conditions. (**a**) Growth curves of WT and ∆PAP strains in Sauton medium supplemented with 1 mM NH_4_Cl. Growth was followed by measuring optical density (OD_600nm_). (**b**) Cells from WT and ∆PAP cultures were labelled with [^14^C]-acetate at the same physiological state: early (T1, OD_600nm_ ~ 0.25), mid-exponential (T2, OD_600nm_ ~ 0.5) and stationary (T3, OD_600nm_ ~ 1.6) phase for 1 h at 37 °C. After organic extraction, ^14^C-labelled lipids were separated by TLC. Optical density standardization was performed for lipid extraction. (**c**) Quantification of the radiolabelling intensity of the TLC showed in panel (**b**) is shown in arbitrary units (AU). The results are the mean values and standard deviation of three independent biological replicates. Solvent system: hexane:diethylether:acetic acid (70:30:1, v/v/v). DAG, Diacylglycerol; TAG, triacylglycerol; WT, *M. smegmatis* mc^2^155; AU, arbitrary units. Statistical significance was calculated using two-way ANOVA test followed by Bonferroni correction; ****P < 0.0001; ***P < 0.001; **P < 0.01; *P < 0.05. The full-length image corresponding to (**b**) is shown in Figure S6.

To evaluate if a compensation effect was arising in the single mutant strains, we constructed a double mutant strain. Disruption of *msPAPα* and *msPAPβ* genes was obtained by a two-step allelic exchange using plasmid pAC20. The correct homologous recombination event was confirmed by PCR (Supplementary Fig. S4) and the resulting strain was named ∆PAP. TAG accumulation was analyzed by total lipid extraction and fractionation by normal-phase TLC. No significant differences in TAG levels were observed when the ∆PAP strain was grown in 7H9 medium (Supplementary Fig. S5). However, under nitrogen-starving conditions, where TAG synthesis is induced, the ∆PAP double mutant presented a longer *lag* phase as well as a delay in entering into the exponential phase. In addition, de novo TAG biosynthesis was analyzed by metabolic labeling with [^14^C]-acetate and a significant reduction in TAG content in early exponential growth phase (T1) was observed (Fig. 3). However, during exponential phase, this phenotype was reverted, and the double mutant strain was able to synthetize even more TAG than the wild type strain.

**Figure 4:**
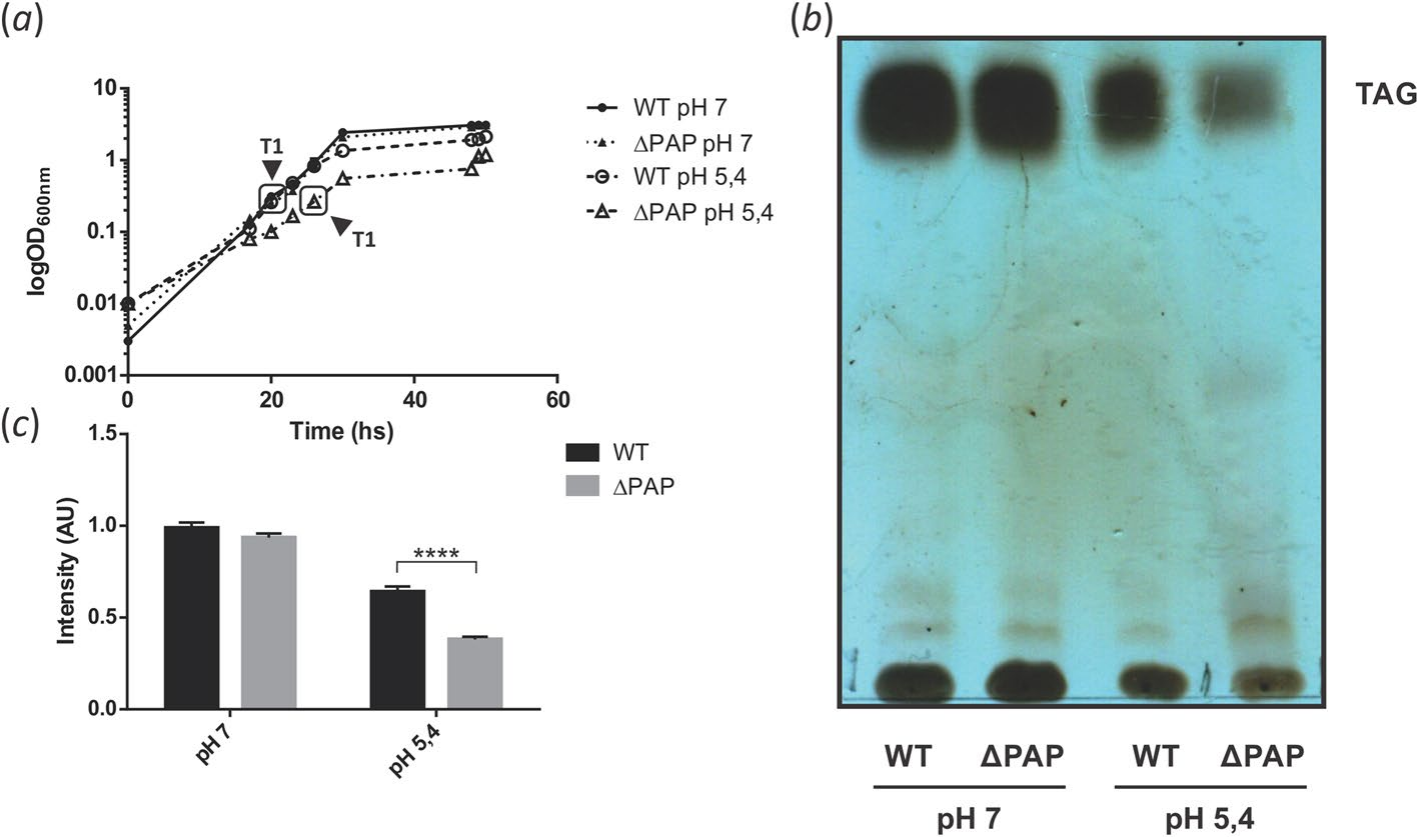
Growth curve and TAG production of *M. smegmatis* ∆PAP at acidic stress. (**a**) Growth curves of WT and ∆PAP strains in 7H9 medium supplemented with glycerol and tyloxapol (pH 7 and 5.4). Growth was followed by measuring optical density (OD_600nm_). (**b**) Lipids from WT and ΔPAP cells were extracted at the same physiological state at early exponential phase (T1, OD_600nm_ ~ 0.25) and then analyzed by TLC. (**c**) Quantification of the intensity of the spots present in the TLC showed in panel (**b**). The results are the mean values and standard deviation of three independent biological replicates. All results were normalized by OD_600nm_ and total protein concentration. Solvent system: hexane:diethylether:acetic acid (70:30:1, v/v/v). TAG, triacylglycerol; WT, *M. smegmatis* mc^2^155; AU, arbitrary units. Statistical significance was calculated using *t-test* analysis ; ****P < 0.0001; ***P < 0.001; **P < 0.01; *P < 0.05.

In order to analyze if the total TAG content was affected under a different stress condition, the wild type and ∆PAP strains were grown in 7H9 medium at pH 7 and pH 5. Our results indicated that the ∆PAP strain was impaired in TAG biosynthesis at acidic pH during early exponential phase (T1) compared with the wild type strain (Fig. 4). These observations support the idea that at pH 7, DAG biosynthesis could arise from alternative pathways complementing the absence of PAP activity.

### Absence of PAP enzymes has a global impact on lipid composition in *M. smegmatis*

**Figure 5:**
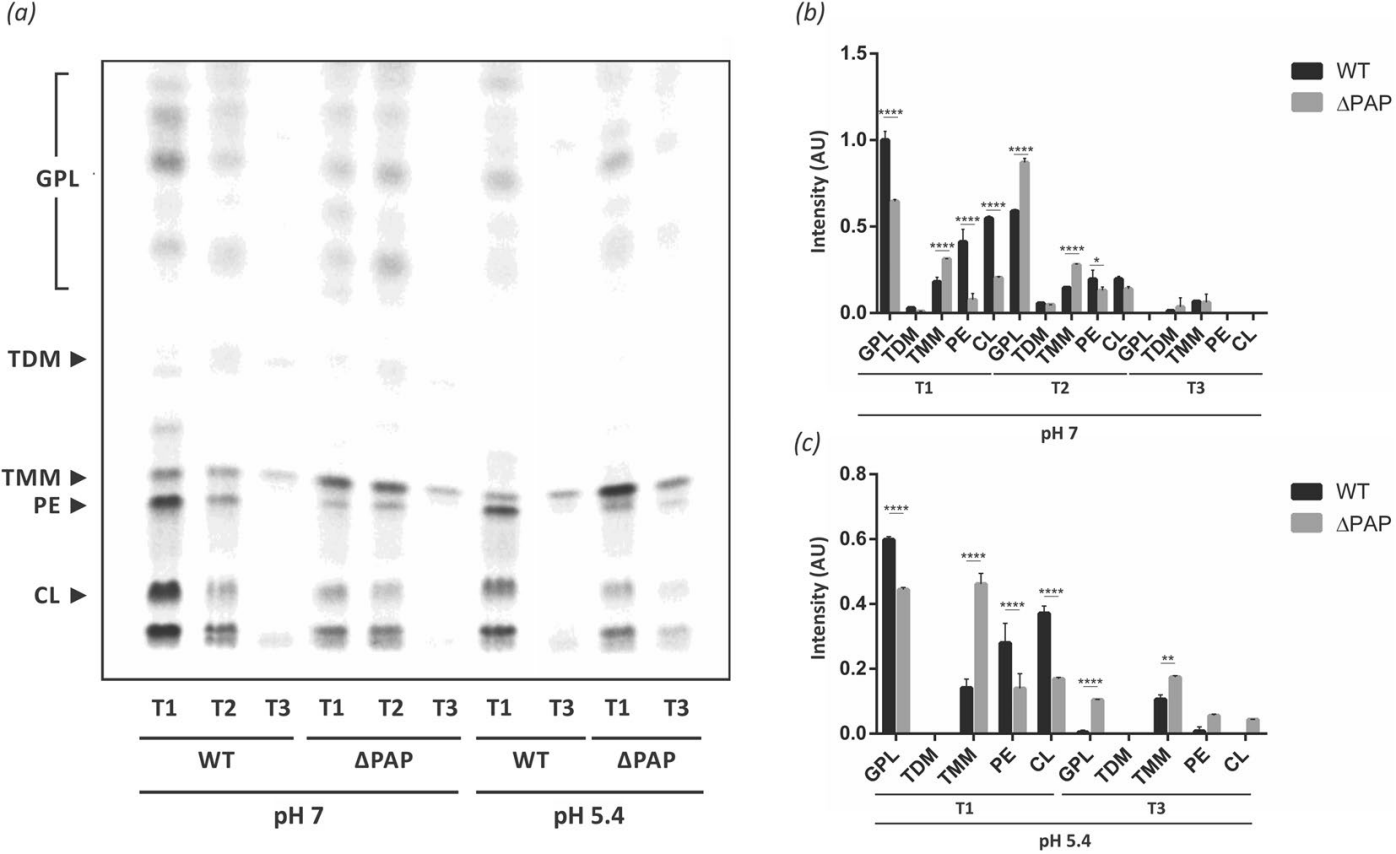
Lipid analysis of the envelope of *M. smegmatis* ΔPAP strain. (**a**) Cells from WT and ∆PAP cultures grown in 7H9 medium (pH 7 and 5.4) were labelled with [^14^C]-acetate at the same physiological state at early (T1, OD_600nm_ ~ 0.25), mid-exponential (T2, OD_600nm_ ~ 0.5) and stationary (T3, OD_600nm_ ~ 1.5) phase for 1 h at 37 °C. Cultures were normalized by OD and after organic extraction; ^14^C-labelled lipids were separated by TLC. (**b**) and (**c**) Quantification of the intensity of the spots of the TLC showed in panel (**a**). The results are the mean values and standard deviation of three independent biological replicates. Solvent system: chloroform:methanol:water (20:4:0,5, v/v/v). GPL, glycopeptidolipids; TDM, trehalose dimycolate; TMM, trehalose monomycolate; PE, phosphatylethanol-amine; CL, cardiolipin; WT, *M. smegmatis* mc^2^155; AU, arbitrary units. Statistical significance was calculated using two-way ANOVA test followed by Bonferroni correction; ****P < 0.0001; ***P < 0.001; **P < 0.01; *P < 0.05.

**Figure 6:**
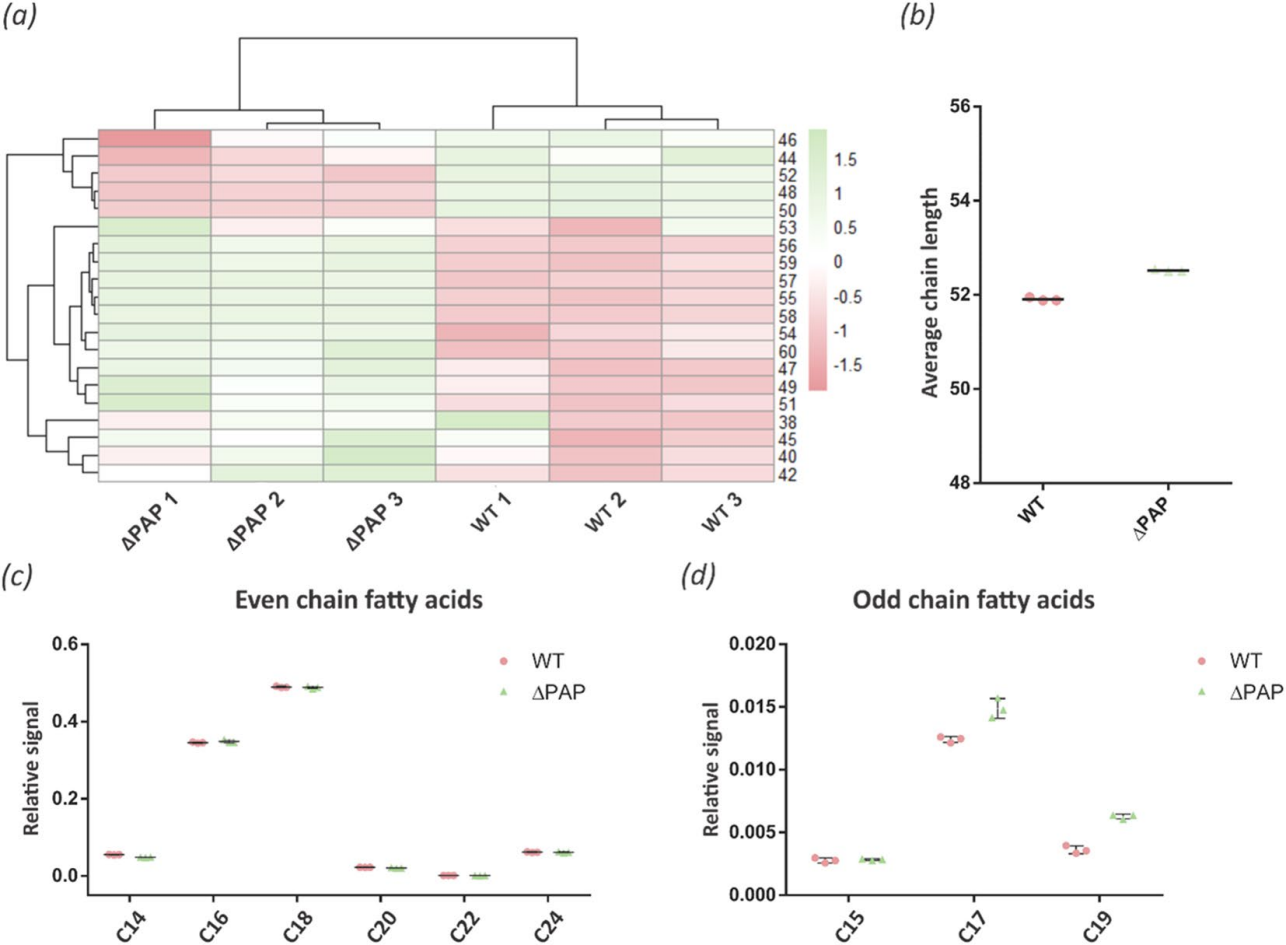
TAG composition of *M. smegmatis* WT and ΔPAP strains grown in 7H9 medium at pH 7. (**a**) Heatmap of the fatty acyl chain length variations in TAG extracted at exponential phase (T2, OD_600nm_ ~ 0.5) from cultures of the WT and ΔPAP strains grown in 7H9 medium at pH 7. (**b**) Weighted average of numbers of carbon in TAG’s chain. (**c**) Relative abundance of TAG with even fatty acid chain. (**d**) Relative abundance of TAG with odd fatty acid chain. WT, *M. smegmatis* mc^2^155 wild type strain. The results are the mean values and standard deviation of three independent biological replicates (WT1, WT2, WT3 and ΔPAP1, ΔPAP2, ΔPAP3).

**Figure 7:**
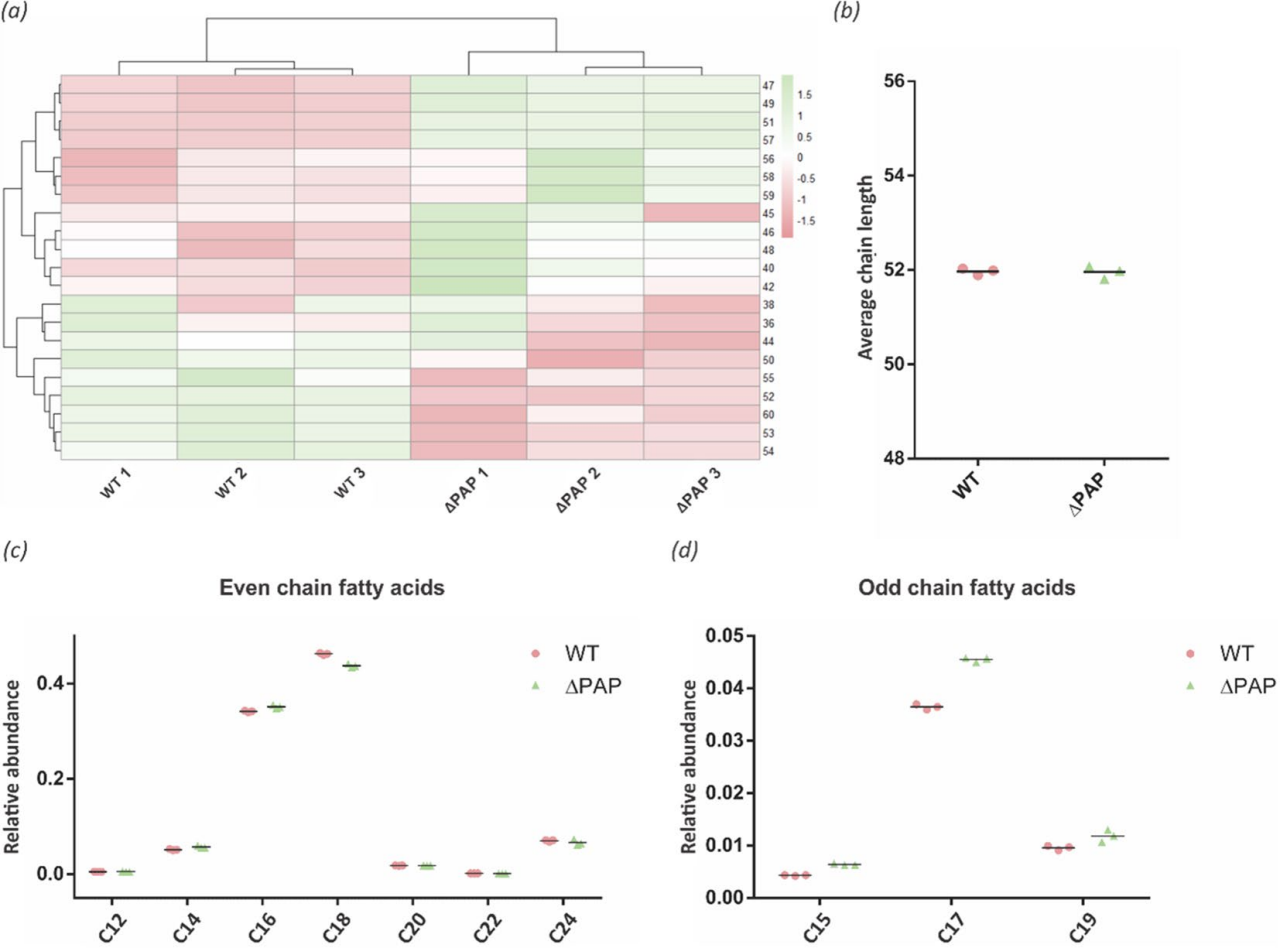
TAG composition of *M. smegmatis* WT and ΔPAP strains grown in 7H9 medium at pH 5. (**a**) Heatmap of fatty acid chain length variations in TAG extracted at exponential growth phase (T2, OD_600nm_ ~ 0.5) from cultures of the WT and ΔPAP strains grown in 7H9 medium at pH 5. (**b**) Weighted average of numbers of carbon in TAG’s chain. (**c**) Relative abundance of TAG with even fatty acid chain. (**d**) Relative abundance of TAG with odd fatty acid chain. WT, *M. smegmatis* mc^2^155 wild type strain. The results are the mean values and standard deviation of three independent biological replicates (WT1, WT2, WT3 and ΔPAP1, ΔPAP2, ΔPAP3).

Bacteria produce glycerophospholipids exclusively through the CDP-DAG-dependent pathway^30,31^, making PA a key metabolic branch point between glycerophospholipid and TAG synthesis (Supplementary Fig. S1). To start evaluating the impact of the deletion of the *pap* genes on glycerophospholipid biosynthesis and on the whole composition of the cell envelope, cultures of the wild type and ∆PAP strains were grown and labeled with [^14^C]-acetate at early exponential, exponential, and stationary growing phases in 7H9 medium at pH 7 and pH 5.4. The lipid content was extracted from each strain and analyzed by TLC. The results shown in Fig. 5 indicated that the ΔPAP strain synthesized reduced levels of glycopeptidolipids and phospholipids (PE and CL) during early exponential phase (T1) at both pH. Additionally, an increase in trehalose monomycolate (TMM) synthesis was observed (Fig. 5). At neutral pH, phospholipid and TMM synthesis was similar in the wild type and ∆PAP strains in the stationary phase of growth (T3). In contrast, TMM synthesis was still higher in the mutant strain at pH 5.4, (Fig. 5). These results suggested that in the absence of PAP activity there is a metabolic rearrangement that redirects the carbon flux towards TAG synthesis in detriment of phospholipids synthesis and prompted us to perform a comparative lipidomic analysis between the ΔPAP and the wild type strains using Mass Spectrometry (MS). Briefly, lipids were extracted from exponential phase cultures of both strains grown in 7H9 medium at pH 7 and pH 5.4. As expected, the ΔPAP strain did not present significant changes in TAG and DAG content at exponential growth phase at both pH analyzed. However, detailed analysis of the fatty acyl composition of TAG revealed that the ΔPAP strain presented a relative increase in the amount of odd-chain FA esterified into TAG compared to the wild type strain (Figs. 6a and 7a), although the weighted average of the number of carbons, both at neutral pH and at acidic pH remained constant in the mutant strain (Figs. 6b and 7b). Furthermore, this difference was specifically related with an increase in fatty acids C15, C17 and C19 (Fig. 6c,d and 7c,d). These results indicated that although the total content of TAG is similar in the wild type and ∆PAP strains, the composition is different, suggesting that the precursors used for the synthesis of TAG in the mutant strain could come from an alternative pathway.

### ∆PAP mutant forms defective biofilms in liquid–air interfaces

**Figure 8:**
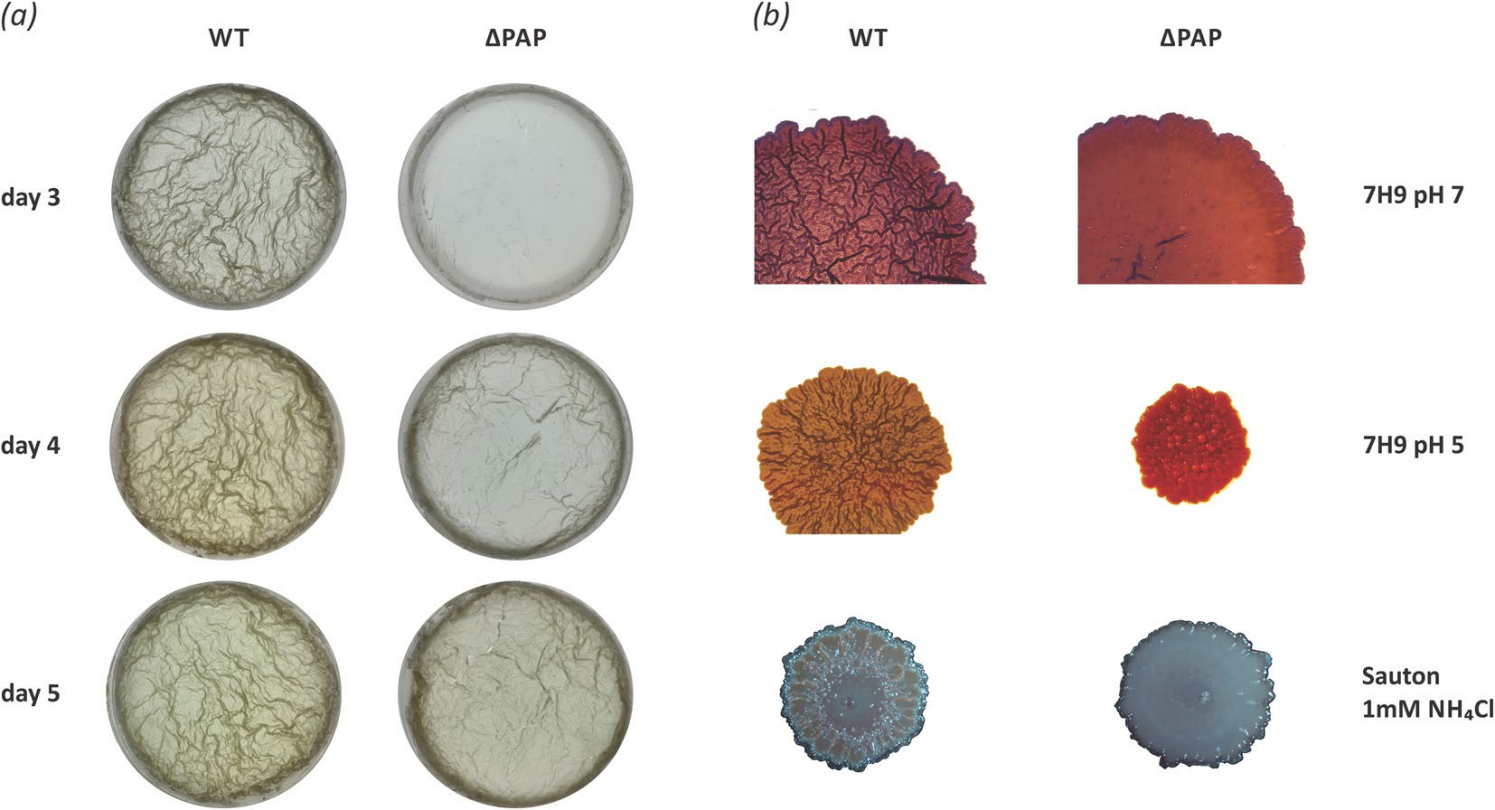
Analysis of biofilms formed in air–liquid interface by the WT and ΔPAP mutant strains. (**a**) WT and ∆PAP strains were incubated in Petri dishes containing a modified version of the M63 medium without shaking at 30 °C for 5 days. (**b**) Colonies of WT and ΔPAP strains grown in 7H9 medium (pH 7 and pH 5.4) and Sauton 1 mM NH_4_Cl during one week at 37° C. The images were acquired with the Olympus MVX10 magnifying glass (0,65X). WT, *M. smegmatis* mc^2^155 wild type strain. The results are representative of three independent biological experiments.

A number of components of the mycobacterial cell wall such as glycopeptidolipids, short chain mycolic acids and monomeromycolyl DAG have been shown to play an important role in formation of pellicle biofilms^32,33^. Even subtle defects in cell wall components can modify the surface properties of individual cells, alter cell-to-cell interactions, and ultimately give rise to colonies with visibly different morphologies^34^. In this regard, we analyzed if the changes in the lipid composition that we observed in the ∆PAP mutant strain had impact on biofilm formation. For this, cultures of the wild type and ΔPAP strains were grown in modified M63 medium^35^ in Petri dishes at 30 °C without shaking. As shown in Fig. 8a, the ∆PAP strain formed defective pellicles. Another way to study biofilm formation is the analysis of macrocolonies. They can present different morphologies, from smooth and opaque to rough and transparent^36^ depending not only on the growth conditions but also in the composition of the extracellular matrix and the cell wall. In this sense, we analyzed colony morphology of the wild type and ∆PAP mutant strain in 7H9-agar medium supplemented with Congo red. The plates were incubated for one week at 37 °C. As shown in Fig. 8b, in 7H9 medium at pH 7 and under nitrogen-starving conditions, the ∆PAP mutant exhibited smooth colony morphology, being unable to form cords in contrast with the opaque and rough colony morphology of the wild type strain. Interestingly, in the presence of acidic stress (pH 5.4) or low concentration of NH_4_^+^, both strains formed smaller colonies than those obtained at pH 7 and the ∆PAP colony was even smaller under acidic stress.

## Discussion

In this work, we describe the identification and physiological characterization of the first PAP enzymes involved in TAG biosynthesis in mycobacteria. By means of a comprehensive bioinformatic analysis of the *M. smegmatis* genome, we were able to identify two candidate genes encoding for putative PAP: *msmeg_0633* and *msmeg_0634*. The functional role of these proteins was analyzed through the heterologous expression in *E. coli.* We observed increased levels of intracellular DAG of up to three and fourfold for MSMEG_0633 and MSMEG_0634, respectively (Fig. 2). Moreover, co-expression of the *S. coelicolor* DGAT enzyme SCO0958 together with MSMEG_0633 in *E. coli* reconstituted the complete pathway for de novo TAG biosynthesis in a heterologous host. Altogether, these results indicated that MSMEG_0633 and MSMEG_0634 are phosphatase enzymes able to catalyze in vivo the formation of DAG from intracellular PA or other analogous lipid of the host cell and we named them msPAPα and msPAPβ.

We found that none of the two *pap* genes were essential for survival of *M. smegmatis* and disruption of either PAPα or PAPβ caused no alteration in the intracellular levels of DAG/TAG. These results suggested that there may be some redundancy in the activity of both enzymes, and that one could complement the deficiency of the other, similar to what had been previously reported in *S. coelicolor*^25^. In this bacterium, growth and DAG/TAG synthesis of the single PAP mutants was indistinguishable from the wild type strain. However, a double mutant strain in the PAP enzymes from *S. coelicolor* showed reduced levels of TAG accumulation in exponential phase, although upon reaching the stationary phase, the levels were restored to that of the wild type strain^25^. Moreover, the effect on gene redundancy had previously been observed in other enzymes that participate in the TAG biosynthesis pathway. This is the case of the Tgs enzymes in *M. tuberculosis*, where a mutant in the *tgs1* gene, whose protein product provides the highest TAG synthase activity, led to a drastic decrease in the synthesis of TAG under conditions of hypoxia, acidic stress and NO. However, traces of TAG synthesis were observed in all these conditions^37^.

We found that *M. smegmatis* mc^2^155 is able to synthesize large amounts of TAGs during the exponential growth phase when grown in 7H9 liquid medium with glycerol as carbon source. At stationary phase we were not able to detect TAG accumulation. However, when *M. smegmatis* was grown in nitrogen limiting conditions, de novo synthesis of TAG was maintained during all stages of growth and this was correlated with a higher accumulation of the reserve compound during the stationary phase. Recently, Santucci et al. reported that the availability of nitrogen and carbon are two factors that influence both the production of TAG and its accumulation in the form of ILI in *M. smegmatis*^38^. However, they found that both in rich medium and in nitrogen-limiting conditions *M. smegmatis* accumulate TAG in stationary phase, and that TAG accumulation was dependent on the concentration of glycerol. The discrepancies with our results could be related with the minimum glycerol concentration used, which was fivefold higher than that used in this work.

Interestingly the double mutant strain ∆PAP, synthesized similar levels of DAG/TAG as the wild type strain (Supplementary Fig. 5S). However, growth and TAG accumulation of the double mutant under nitrogen limiting conditions or acidic stress showed several differences both in growth rate and TAG biosynthesis (Figs. 3 and 4). Moreover, the ΔPAP strain synthesized reduced levels of glycopeptidolipids and phospholipids and increased levels of TMM. While TMM is most likely the form under which mycolic acids are exported to the cell wall and outer membrane, glycopeptidolipids are present in the outer leaflet of *M. smegmatis* and are important in biofilm formation, aggregation, motility and cell wall integrity^39^. Altogether, the results suggest that under stress conditions other lipid biosynthetic pathways are altered in order to provide DAG for TAG biosynthesis (Fig. 5). Besides PAP enzymes, there are different alternatives for the production of DAG in bacteria. The experience with the *E. coli* model suggests that DAG accumulation is deleterious to bacterial growth and DAG kinases are key enzymes in lipid metabolism that phosphorylate DAG formed by the turnover of membrane phospholipids. For example, in *E. coli*, the phospholipid phosphatidylglycerol (PG) is degraded to DAG by the transfer of the glycerol-1-phosphate head group to membrane-derived oligosaccharides (MDO), that function in osmotic homeostasis^40,41^. In this case, the DAG formed is converted to phosphatidic acid for the resynthesis of PG by the enzyme DgkA^42^. In gram positive bacteria like *B. subtilis*; PG is the source of the glycerol-1-phosphate groups needed for lipoteichoic acid (LTA). LTA is a zwitterionic polymer found in the cell wall of many Gram-positive bacteria, consisting of a polyglycerolphosphate (PGP) chain that is linked via a glycolipid anchor to the bacterial membrane^43^. PG turnover is rapid in these bacteria and the large amount of DAG formed requires a DagK for its efficient reintroduction into the phospholipid biosynthetic pathway^44^. It is interesting to note that recent literature has highlighted the presence of a PGP-type LTA in various Actinobacteria. However, these bacteria appear to lack LTA synthase enzyme (LtaS) homologues and, therefore, it has been suggested that the polymer is synthesized by an alternative pathway^45^. Interestingly, although the double mutant strain accumulated similar levels of DAG/TAG compared to the wild type strain, detailed analysis of TAGs indicated that the ΔPAP strain presented a relative increase in the amount of odd-chain TAG compared to the wild type strain. This difference was specifically related to increased levels of C15, C17 and C19 fatty acids in the TAG of the mutant strain (Figs. 6 and 7). Mycobacteria contain high levels of methyl branched fatty acids within the cell envelope^46,47^, being the most abundant the tuberculostearic acid^48^. This fatty acid consists of a C19 monomethyl-branched stearic acid and is mainly found in phospholipids, attached by covalent bonds to lipid virulence determinants such as phosphatidylinositolmannosides (PIMs), lipoarabinomannan (LAM), and related cell wall glycolipids^49^. Altogether, these observations support an active role for the PAP enzymes during stress and strongly suggest that carbon flux in the ∆PAP mutant is being redirected from the synthesis of phospholipids towards the synthesis of TAG.

The ability of environmental mycobacteria to form biofilm has been widely described in many mycobacteria species including *M. avium*^50^, *M. fortuitum*^51^, *M. marinum*^52^ and *M. smegmatis*^32^. It was also observed in subsequent studies that some mycobacteria can develop structures not only on surfaces, but also at the air–liquid interface^33,53^. This phenomenon can be explained by the composition of the extracellular matrix of the biofilm and the unique characteristic of the mycobacterial cell wall, especially the presence of a large number of lipids. Furthermore, it has been observed that *M. smegmatis* is able to form well organized colonies which have been described as a type of biofilm. These colonies are composed of cells encapsulated by a large amount of exopolysaccharides^54^. In both cases, the components of the cell wall play a central role both in the initial formation of the biofilm^32^ as well as in its maturation^55^. Additionally, high amounts of DAG and TAG have been found in the outer membrane of *M. smegmatis*^56^ but it is unknown if they play a role in the formation of these structures. In this work we found that the ∆PAP strain formed a defective biofilm and exhibited smooth colony morphology in contrast with the opaque and rough colony morphology of the wild type strain (Fig. 8). In mycobacteria, mycolic acids can also be found as mycolyl-diacylglycerols (MDAG), in which a mycolic acid (or mycolate) is esterified to a glycerol moiety. Based on our results, one possible explanation is that upon PAP depletion, the DAG necessary for TAG biosynthesis could arise from the recycling of MDAG. Chen et al. showed that reduction of MDAG biosynthesis resulted in the accumulation of TAGs, suggesting a metabolic relationship between these two molecules^57^. MDAG play a role in colony morphology and biofilm formation of mycobacteria. Loss of hydrophobic MDAGs is consistent with a smooth colony phenotype on solid media and defect in pellicle formation in liquid media.

It is known that during infection *M. tuberculosis* faces variable environments. For instance, the pH is highly dynamic and can fluctuate depending on the activation state of macrophages^58^ or within the granulomas, with a mean value of pH 5.5^59^. Deb et al. observed that when acidic stress is combined with other types of stress such as hypoxia or low availability of nutrients, *M. tuberculosis* accumulates large amounts of TAG^6^. However, the effect of acidic stress has not been described either in the ability to form macrocolonies or in the synthesis of TAG in *M. smegmatis*. In this study we found that both, the wild type and ∆PAP mutant strains formed smaller colonies when they were grown under stress conditions such as limiting nitrogen concentration or acidic pH (Fig. 8).

The identification of msPAPα and msPAPβ completes the minimal set of enzymes together with the redundant set of DGATs previously characterized^13,17,37,60,61^ required for de novo TAG biosynthesis in mycobacteria. ILI formation in *M. tuberculosis* has been described as an adaptation strategy promoting survival during periods of non-replicating persistence in vitro but also in vivo, mainly regulated by the dormancy regulon^18,62^. Overall, our results provide new elements for the study of TAG biosynthesis and regulation in mycobacteria.

## Methods

### Bacterial strains, culture, and transformation conditions

The strains and plasmids used in this study are described in Supplementary Tables S1 and S2. The *E. coli* strain DH5α^63^ was used for routine sub-cloning and was transformed according to Sambrook et al.^64^. *E. coli* strains were grown in Luria–Bertani (LB) medium at 37 °C and supplemented when needed with the following antibiotics: 100 µg ml^−1^ amplicillin (Ap), 50 µg ml^−1^ kanamycin (Km), 20 µg ml^−1^ chloramphenicol (Cm) or 100 µg ml^−1^ hygromycin (Hyg). *M. smegmatis* mc^2^155 is an electroporation-efficient mutant of mc^2^6^65^. Liquid cultures of *M. smegmatis* mc^2^155 (WT), ∆PAPα and ∆PAPβ, and ∆PAP were grown at 37 °C in Middlebrok 7H9 or modified Sauton medium containing (g l^−1^) K_2_HPO_4_, 0.5; MgSO_4_, 0.5; ferric citrate, 0.05 g; ZnSO_4_, 0.001; citric acid, 2, supplemented with 0.2% glycerol and 0.03% Tyloxapol. When required, gentamicin 20 mg ml^−1^ was added to the medium.

Cultures were started with 1/200 dilutions from pre-made cultures with an initial OD of 1; except for the experiment shown in Fig. 4 where 1/100 dilutions were used for the cultures grown at pH 5.4.

For biofilm cultures grown on liquid medium, 10 ml of biofilm medium (a modified version of M63^35^) was inoculated with 10 ml of a saturated culture in a 60 × 15 mm^2^ polystyrene Petri dish and incubated at 30 °C (unless otherwise stated) without disturbance.

### DNA manipulation, plasmid construction and mutant generation

Isolation of plasmid DNA, restriction enzyme digestion and agarose gel electrophoresis were carried out by conventional methods^64^. Genomic DNA of *M. smegmatis* was obtained as described previously^66^.

#### pAC6

*msmeg_0634* was PCR-amplified from genomic DNA of *M. smegmatis* mc^2^155 using the oligonucleotides ACA-003 to introduce an *Nde*I site at the translational start codon of *msmeg_0634* gene, and ACA-004 to introduce an *Eco*RI site at the end of the ORF. To generate a *msmeg_0634* His tag fusion gene, the PCR product was digested with *Nde*I and *Eco*RI and cloned into *Nde*I/*Eco*RI cleaved pET-28a(+), yielding pAC5. Finally, pAC5 was digested with *Xba*I and *Hind*III and cloned into *Xba*I/*Hind*III cleaved pBAD33, yielding pAC6.

#### pAC17

For the construction of the *M. smegmatis* ∆PAPβ mutant strain, the upstream region of *msmeg_0634* gene was amplified with the primers ACA-005 and ACA-006. The 866 bp PCR product was cloned into pCR BluntII TOPO (Invitrogen) yielding pAC2 plasmid. The downstream region of *msmeg_0634* gene was amplified with the primers ACA-007 and ACA-008. The 1210 bp PCR product was cloned into pCR BluntII TOPO (Invitrogen) yielding plasmid pAC3. Plasmids pAC2 and pAC3 were digested with *Xba*I/*Nde*I or *Nde*I/*Spe*I, respectively, and the fragments obtained were cloned into *Spe*I cleaved pPR27 plasmid, yielding pAC17.

#### pAC20

For the construction of the *M. smegmatis* ∆PAP mutant strain the upstream region of *msmeg_0633* gene was amplified with the primers ACA-013 and ACA-014. The 942 bp PCR product was cloned into pGEM-T Easy (Promega) and digested with *Nde*I/*Spe*I. The fragment was inserted into *Nde*I/*Spe*I cleaved pAC2, yielding pAC19. Finally, the pAC19 was digested with *Spe*I and the ∆PAP fragment was inserted into *Spe*I cleaved pPR27 plasmid, yielding pAC20.

#### pAC21

For the construction of the *M. smegmatis* ∆PAPα mutant the upstream region of *msmeg_0633* gene was amplified with the primers ACA-013 and ACA-014 and the downstream region with the primers ACA-015 y ACA-016. The 942 pb and 1069 pb PCR products, were cloned into pGEM-T Easy (Promega) yielding pAC15 and pAC16 plasmids, respectively. pAC15 was digested with *Pst*I/*Nde*I and the 966 pb fragment was cloned into *Pst*I/*Nde*I cleaved pAC16, yielding pAC18. Finally, pAC18 was digested with *Spe*I and the ∆*ms0633* fragment was inserted into *Spe*I cleaved pPR27, yielding pAC21.

#### pAC35

*msmeg_0633* was PCR-amplified from genomic DNA of *M. smegmatis* mc^2^155 using the primers ACA-011 to introduce an *Nde*I site at the translational start codon of *msmeg_0633* gene, and ACA-012 to introduce an *Eco*RI site at the end of the ORF. To generate a *msmeg_0634* His tag fusion gene, the PCR product was digested with *Nde*I and *Eco*RI and cloned into *Nde*I/*Eco*RI cleaved pET-28a(+), yielding pAC5. Finally, pAC5 was digested with *Xba*I and *Hind*III and cloned into *Xba*I/*Hind*III cleaved pBAD33, yielding pAC6.

### Construction of ∆PAPα, ∆PAPβ and ∆PAP double mutant strains of *M. smegmatis*

For the construction of the *M. smegmatis* mutants ∆PAPα*,* ∆PAPβ*,* and ∆PAP the plasmids pAC21, pAC17 or pAC20 were used to transform *M. smegmatis* cells. Gm-resistant and XylE^+^ transformants were analyzed by colony PCR and those that presented a legitimate recombination event in the chromosomal copy of the respective gene were selected and grown at 37 °C and plated at 42 °C in the presence of sucrose to promote a second recombination event, resulting in the replacement of the wild type copy of the genes for the truncated versions. The correct double recombination events present in the mutant strains were confirmed by PCR.

### Recombinant protein expression in *E. coli* and lipid analysis

For the expression of heterologous proteins in *E. coli*, the GG-02 and GG-03 strains derived from MPS11 were grown in LB media at 37 °C until DO_600nm_ 0.6. PAP protein expression was induced by addition of l-arabinose 0.2% and cultures were grown overnight at 23 °C. For SCO0958-MS0633 or SCO0958-MS0634 co-expression experiments, cells at OD_600nm_ 0.6 were induced by l-arabinose 0.2% and IPTG 0.1 mM and grown overnight at 23 °C. For lipid analysis, cultures were incubated for 1 h with 3 µCi of [^14^C]-acetate and total lipids were extracted as described by Bligh & Dyer^67^.

The lipid extracts were dried and analyzed by TLC on silica gel 60 F254 plates (0 ± 2 mm, Merck), using the solvent system hexane/diethylether/acetic acid (70:30:1, v/v/v)^68^. For ^14^C labeled lipids the radioactivity incorporated into each lipid fraction was quantified using Typhoon FLA 7000 (GE Healthcare) and the corresponding spots were quantified using ImageJ software (version 1.52a).

### Lipids analysis

#### Lipid analysis using thin layer chromatography (TLC)

For lipid extraction, the volume of each sample was normalized by OD_600nm_, e.g. 10 ml of a culture of OD_600nm_ 0.5 was collected by centrifugation and lipids were extracted as described by Bligh & Dyer^67^. The lipid extracts were dried and resuspended in 50 µl of chloroform and 10 µl were analyzed by TLC on silica gel 60 F254 plates (0 ± 2 mm, Merck). For TAG analysis, the solvent system used was hexane/ diethylether/acetic acid (70: 30: 1, v/v/v) and for phospholipid and cell wall component analyses the solvent system was chloroform/methanol/water (20:4:0.5, v/v/v). Lipid fractions were visualized by Cu-phosphoric staining and identified by comparing to the mobility of known standards. For ^14^C labeled lipids, the radioactivity incorporated into each lipid fraction was quantified using Typhoon ™ FLA 7000 laser scanner (GE Healthcare) and the corresponding spots were quantified using ImageJ software (version 1.52a).

#### LC–MS-MS lipid analysis

Lipids were extracted from 2 mg of lyophilized cells using a biphasic separation with MTBE, methanol and water^69^. Briefly, 500 µl of ice-cold methanol were added to the cells and the mixture was vortexed for 20 s. Then, 2000 µl of methyl tert-butyl ether (MTBE) were added and the mixture was incubated under agitation for 30 min at 4 °C. After addition of 500 µl of water, samples were vortexed for 1 min and centrifuged at 14,000×*g* for 10 min at room temperature. The upper phase containing the lipids was collected and dried down under nitrogen. The dry extracts were reconstituted with 300 μL of 9:1 methanol:toluene with 10 mM of ammonium acetate and centrifuged at 14,000×*g* for 5 min before analysis by MS. Water extracted using the same protocol was used as a blank control.

Flow injection analysis (FIA) was performed at 7 μl min^−1^ using a Nexera X2 UHPLC system (Shimadzu) and 1:1 dichloromethane:methanol with 10 mM of ammonium acetate as running solution. Lipids were analyzed using a QTRAP 5500 mass spectrometer (Sciex).

Lipid molecular species were relatively quantified by MRM in positive and negative ionization mode. Lipid classes were quantified using the following ion forms: [M+NH4] + DAG, MAG and TAG, [M−H]− for phospholipids and FFA. Source and gas setting were as follow: Curtain Gas = 17, CAD Gas = Medium, Ion Spray Voltage = 4.1 kV in positive mode and − 2.5 kV in negative mode, Temperature = 200 °C, Nebulizing Gas = 17 and Heater Gas = 25.

### Statistical analysis

The statistical analyses were made using GraphPad Prism software. Data are reported as arithmetic means of the results obtained from three independent experiments ± standard deviations. Statistical significance was calculated using *t-test* or ANOVA test followed by Bonferroni correction as described in the figure legends. Statistical significance was accepted at *p* < 0.05.

Curve fitting was done using the following equation and parameters:

$$ {\text{Four - parameter}}\;{\text{logistic}}\;{\text{curve}}(4\;{\text{PL}}):{\text{Y}} = {{{\text{OD}}0 + ({\text{ODf}} - {\text{OD}}0)} \mathord{\left/ {\vphantom {{{\text{OD}}0 + ({\text{ODf}} - {\text{OD}}0)} {\left( {1 + 10^{{\left( {Log(t1/2) - X*{\text{HillSlope}}} \right)}} } \right)}}} \right. \kern-\nulldelimiterspace} {\left( {1 + 10^{{\left( {Log(t1/2) - X*{\text{HillSlope}}} \right)}} } \right)}} $$

Parameters: t_1/2_ is the time that gives an OD at half way between OD_0_ (initial OD) and OD_f_ (final OD).

## Supplementary Information

Supplementary Information.
