# Supplementary material for: Characterization of key enzymes involved in triacylglycerol biosynthesis in mycobacteria
Source: Sci Rep. 2021 Jun 24;11:13257. doi: 10.1038/s41598-021-92721-y (PMC8225852; doi:10.1038/s41598-021-92721-y)
Supplement: Supplementary file 1 — Supplementary Information. [file 41598_2021_92721_MOESM1_ESM.pdf]

Supplementary information

CHARACTERIZATION OF KEY ENZYMES INVOLVED IN TRIACYLGLYCEROL BIOSYNTHESIS IN MYCOBACTERIA

Crotta Asis, A.[1], Savoretti, F.[1], Cabruja, M [1#], Gramajo, H.[1*], Gago, G.[1*]

TABLE S1. PLASMIDS USED IN THIS STUDY

Name	Description and features	Reference
pET-0958	pET28a(+) with *SCO0958* His tag fusion gene, under the control of T7 promoter, Kan^R	[1]
pAC6	pBAD33 with *msmeg_0634* His tag fusion gene, under the control of P_{BAD} promoter, Cm^R	This study
pAC17	Vector for ΔPAPβ mutant construction, *sacB*, *xylE*, Gm^R	This study
pAC20	Vector for ΔPAP mutant construction, *sacB*, *xylE*, Gm^R	This study
pAC21	Vector for ΔPAPα mutant construction, *sacB*, *xylE*, Gm^R	This study
pAC35	pBAD33 with *msmeg_0633* His tag fusion gene, under the control of P_{BAD} promoter, Cm^R	This study

Kan^R, kanamycin resistance; Cm^R, chloramphenicol resistance; Gm^R, gentamycin resistance

TABLE S2. STRAINS USED IN THIS STUDY

Name	Description or Genotype	Reference
DH5α	*E. coli K12 F-ΔlacU169 (ϕ80lacZΔM15) endA1 recA1 hsdR17 deoR supE44 thi-λ- gyrA96 relA1*	[2]
MPS11	*E. coli* BL21 (DE3) Δ*dgkA*/pET-0958,	[3]

	Kan^R	
GG-02	MPS11 carrying pAC6 plasmid, Kan^R, Cm^R	This study
GG-03	MPS11 carrying pAC35 plasmid, Kan^R, Cm^R	This study
M. smegmatis mc^2155	*M. smegmatis* electroporation-efficient mutant strain of mc26	[4]
ΔPAPα	*M. smegmatis* mc^2155 background. Deletion of *msmeg_0633* gene	This study
ΔPAPβ	*M. smegmatis* mc^2155 background. Deletion of *msmeg_0634* gene	This study
ΔPAP	*M. smegmatis* mc^2155 background. Deletion of *msmeg_0633* and *msmeg_0634* genes	This study

TABLE S3. OLIGONUCLEOTIDES USED IN THIS STUDY

Name	Sequence (5'-3')
ACA-03 (MS0634_fw)	CATATGGATGGTCGGGTGGAGAGAAC
ACA-04 (MS0634_rv)	GAATTCGGTCGGCGAGCCCTACAC
ACA-05 (MS0634_up_fw)	GATATCGCGAGGGCGAGCACAGC
ACA-06 (MS0634_up_rv)	CATATGGCCGACGCGATCAGCAACC
ACA-07 (MS0634_down_fw)	CATATGACGTCAGGGCAAGGGGCGAA
ACA-08 (MS0634_down_rv)	ACTAGTCGCCCCGACCGCAGCATCAT
ACA-11 (MS0633_fw)	CATATGGACGTGCCAAAGATCGGC
ACA-12 (MS0633_rv)	CAATTGACGTCAGGGCAAGGGGC
ACA-13 (MS0633_up_fw)	CTCGAGAGGGCTACGACCGCACCTAT
ACA-14 (MS0633_up_rv)	CATATGCTTCGGCATGAGGGAGTC
ACA-15 (MS0633_down_fw)	CATATGGAGGTCGGCGAGCCCTAC
ACA-16 (MS0633_down_rv)	ACTAGTGGTCACCGCCCCGTTCTG
ACA-17	GACAGGATCTGCGGAAGTGT
ACA-18	GAGCCTGGTGACCAATCAGA

TABLE S4. RAW DATA, INDICATING THE RELATIONSHIP BETWEEN OD AND TIME OF INCUBATION FOR FIGURE 3

Time (hs)	WT			ΔPAP		
0	0.005	0.005	0.005	0.005	0.005	0.005
15	0.22	0.21	0.22	0.11	0.11	0.11
18	0.26	0.24	0.27	0.13	0.13	0.14
21	0.63	0.49	0.52	0.24	0.23	0.22
23	0.74	0.65	0.72	0.31	0.3	0.29
25	0.91	0.91	0.88	0.47	0.42	0.45
28	1.09	0.95	1.04	0.58	0.51	0.62
45	1.59	1.42	1.59	1.57	1.6	1.57
48	1.58	1.28	1.6	1.57	1.59	1.58
50	1.6	1.27	1.58	1.6	1.63	1.62

TABLE S5. RAW DATA, INDICATING THE RELATIONSHIP BETWEEN OD AND TIME OF INCUBATION FOR FIGURE 4

Time (hs)	WT pH 7			DPAP pH 7			WT pH 5,4			DPAP pH 5,4		
0	0.003	0.003	0.003	0.005	0.005	0.005	0.01	0.01	0.01	0.01	0.01	0.01
17	0.13	0.12	0.13	0.15	0.16	0.16	0.11	0.11	0.11	0.15	0.16	0.16
20	0.3	0.31	0.32	0.25	0.23	0.26	0.27	0.25	0.26	0.13	0.12	0.13
23	0.513	0.45	0.45	0.4	0.35	0.38	0.51	0.48	0.45	0.15	0.17	0.15
26	1.078	0.81	0.81	0.84	0.89	0.93	0.82	0.81	0.85	0.23	0.21	0.23
30	2.3	2.45	2.52	2.2	1.98	2.1	1.3	1.45	1.32	0.52	0.47	0.48
48	3.23	2.86	3.07	2.81	3.1	2.75	1.85	2.05	1.85	0.73	0.69	0.7
47	3.13	3.12	3.01	3.15	2.92	3.2	1.91	2.04	1.91	0.9	0.92	0.96
50	3.15	3.2	2.9	3.01	3.1	3.2	2	2.15	2.23	1.08	0.99	1.25

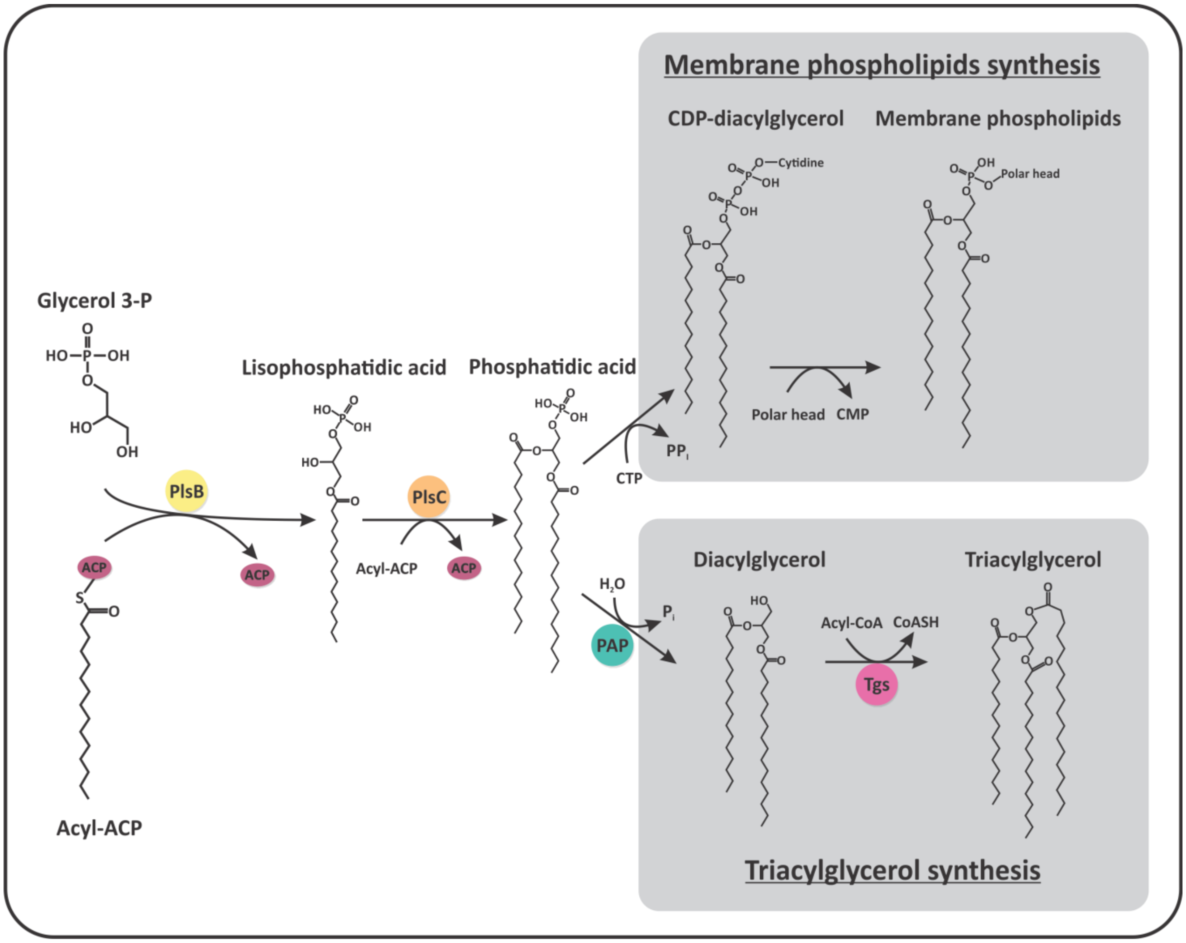

Figure S1. Biosynthesis of membrane glycerophospholipids and triacylglycerols in mycobacteria. Phosphatidic acid is the metabolic branch point in-between glycerophospholipid and triacylglycerol biosynthesis.

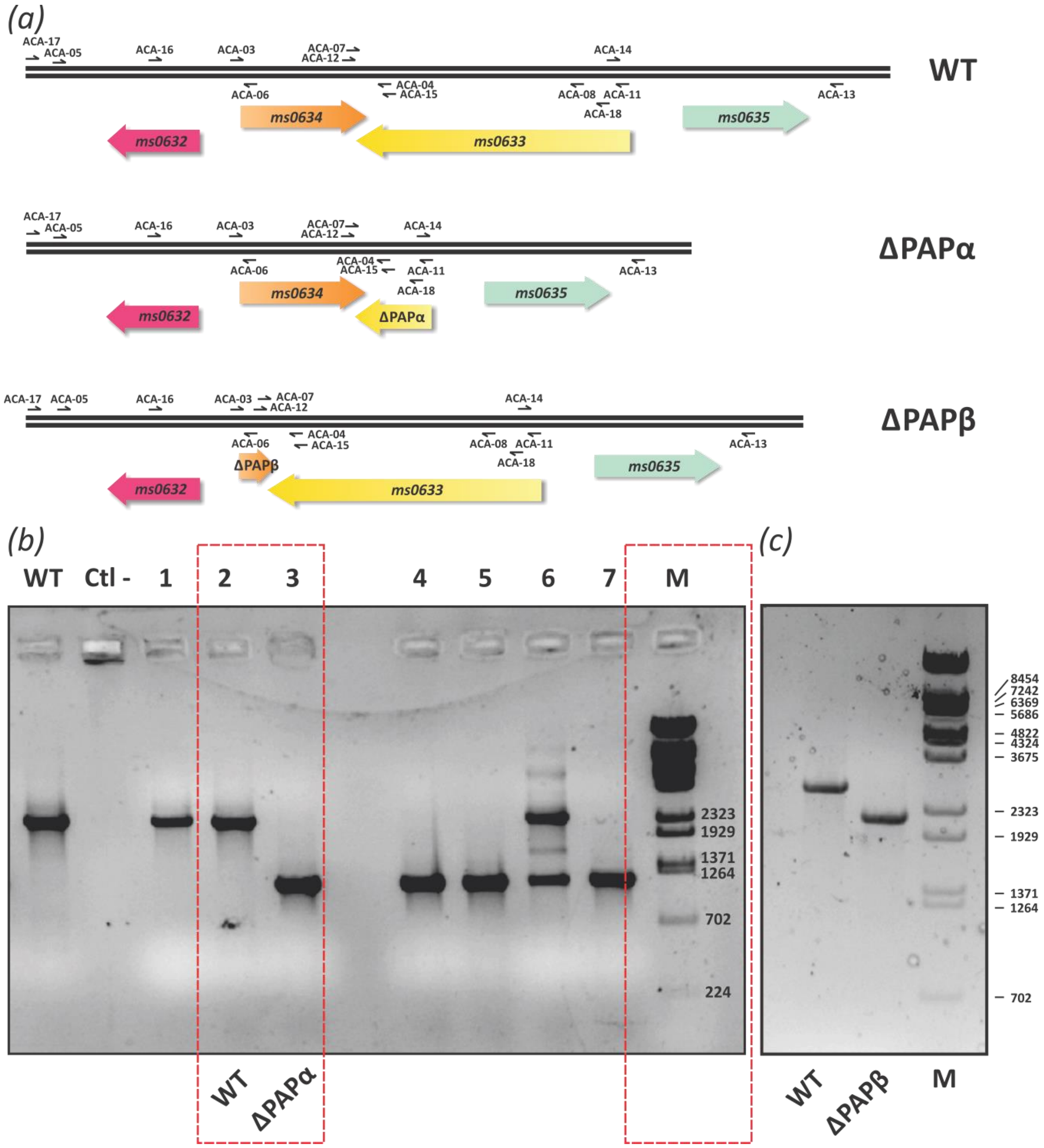

Figure S2. Construction of PAP mutants in *M. smegmatis*. *(a)* Schematic representation of *M. smegmatis* wild type (WT), ΔPAPα and ΔPAPβ strains. Deletion of the target gene was verified by PCR using genomic DNA of the *M. smegmatis* mutant strains. *(b)* PCR amplification of genomic DNA obtained from several mutant candidates (lanes 1-7) and from the WT strain using the specific primers ACA-011 and ACA-016 (Expected size of PCR amplification products: WT: 2452 pb; ΔPAPα*:* 1178 pb). *(c)* PCR amplification of WT and ΔPAPβ genomic DNA using the specific primers ACA-17 and ACA-18 (Expected size of PCR amplification products: WT: 2794 pb; ΔPAPβ*:* 2212).

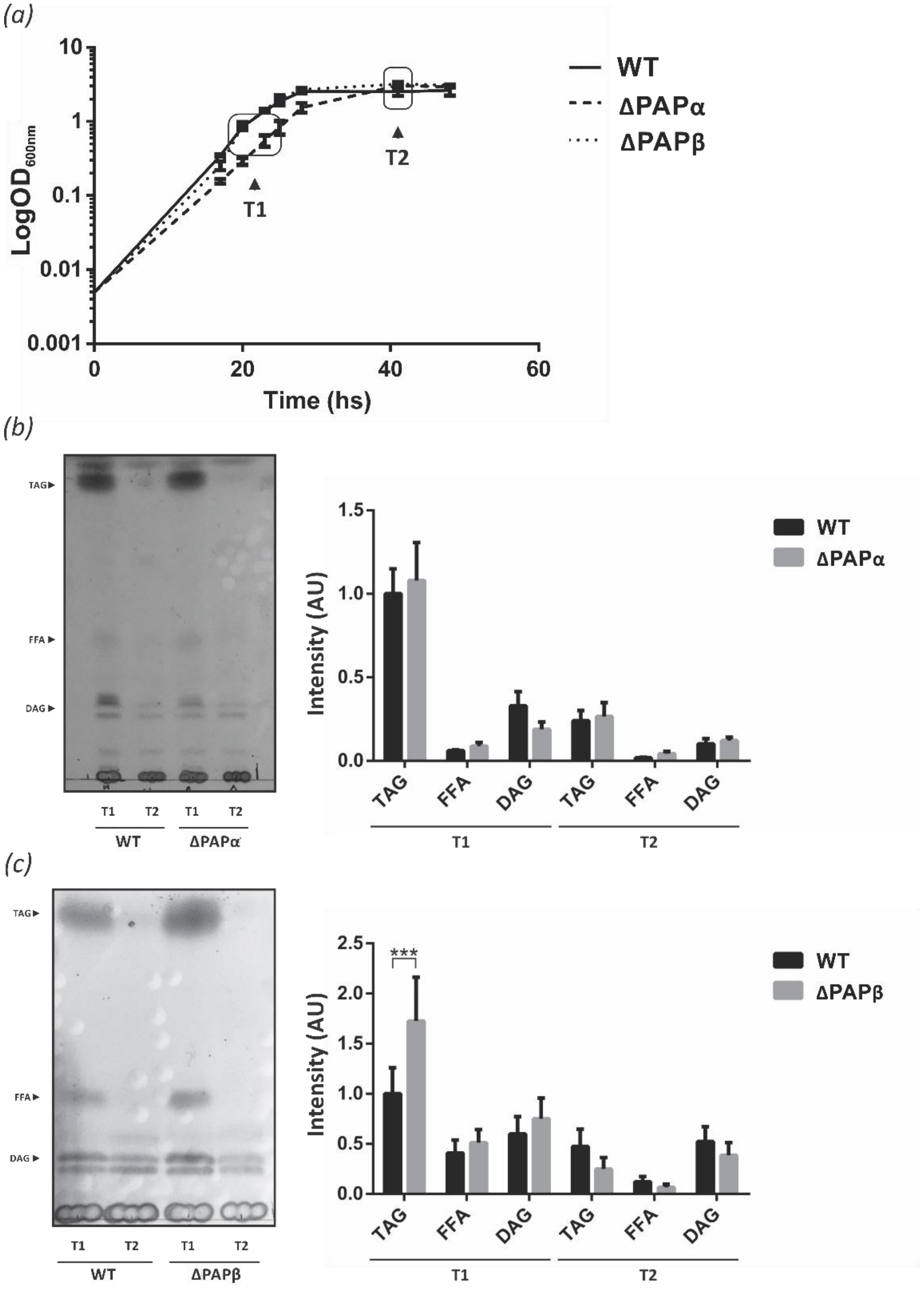

Figure S3. Analysis of *M. smegmatis* ΔPAPα and ΔPAPβ strains. *(a)* Growth curve of ΔPAPα, ΔPAPβ and wild type strains in 7H9 medium supplemented with glycerol and tyloxapol. Growth was followed by measuring optical density (DO_{600nm}). Lipid extracts were analyzed from *M. smegmatis* mc^{2}155 strain and ΔPAPα *(b)* or ΔPAPβ *(c)* on silica gel TLC plates and developed in hexane: diethylether:acetic acid (70:30:1, v/v/v). Aliquots were separated to analyze the lipid profile during exponential (T1, OD_{600nm}~ 0.5) and

stationary (T2, OD_{600nm}~ 1.5) phase. Quantification is shown for both TLC in arbitrary units (AU). The results are the mean values and standard deviation of three independent biological replicates. All results were normalized by OD_{600nm}. TAG, triacylglycerol; FFA, free fatty acids; DAG, Diacylglycerol; WT, *M. smegmatis* mc^2155. Statistical significance was calculated using two-way ANOVA test followed by Bonferroni correction; ***, $P < 0,001$.

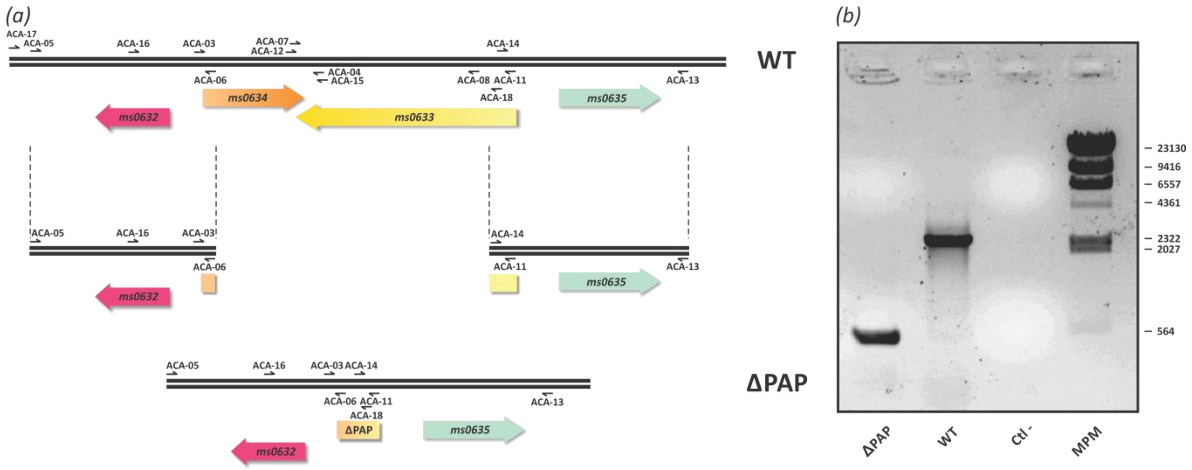

Figure S4. Construction of double deletion mutant lacking *msmeg_0633* and *msmeg_0634* in *M. smegmatis*. *(a)* Schematic representation of *M. smegmatis* mc^{2}155 and ΔPAP strains. *(b)* The genetic organization of the *M. smegmatis* ΔPAP mutant strain was verified by PCR. The amplification was performed using a representative clone and the specific primers ACA-11 and ACA-16 that hybridize outside possible recombination sites (WT: 2452 pb; ΔPAP: 504 pb). WT, *M. smegmatis* mc^{2}155.

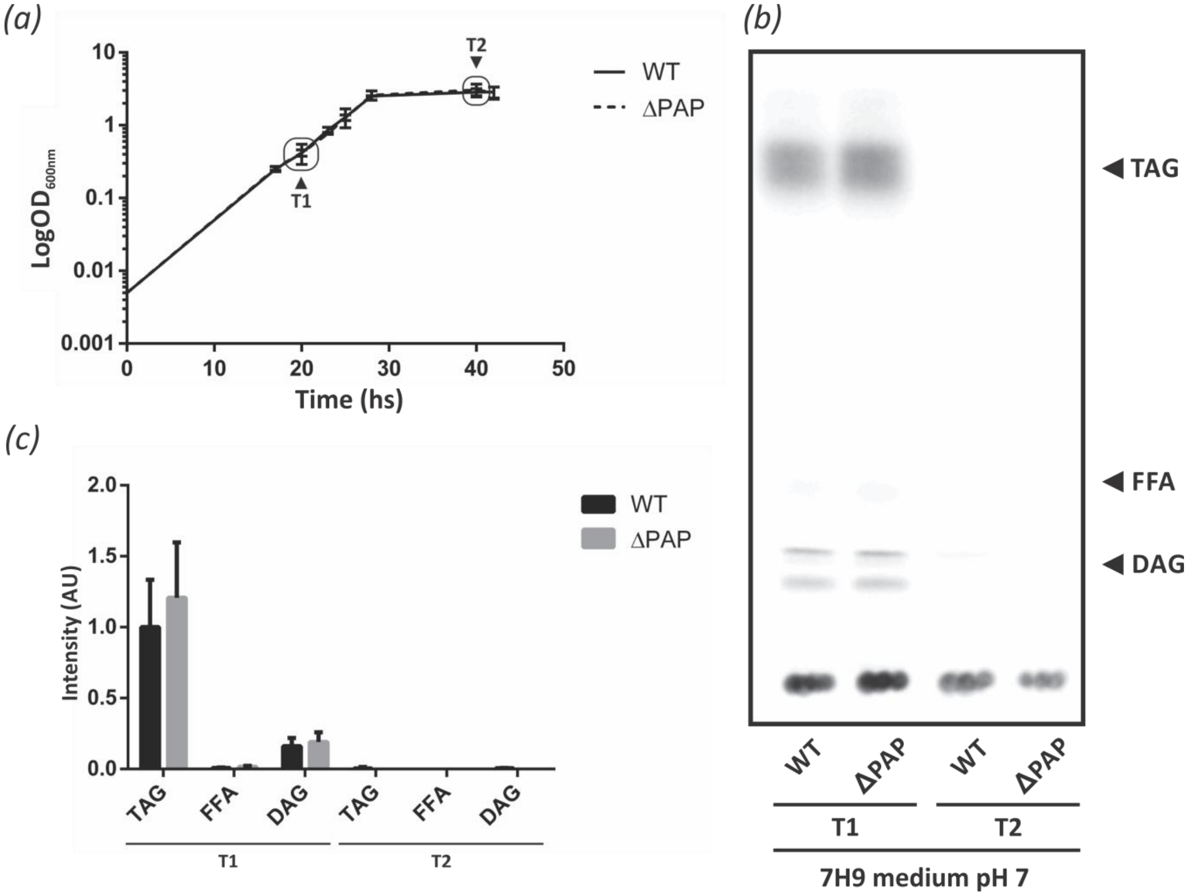

Figure S5. Analysis of *M. smegmatis* ΔPAP strain. *(a)* Growth curve of ΔPAP and wild type strains in 7H9 medium supplemented with glycerol and tyloxapol. Growth was followed by measuring optical density (DO_{600nm}). Aliquots were separated to analyze the lipid profile during exponential (T1, OD_{600nm}~ 0.4) and stationary (T2, OD_{600nm}~ 2.5) phase. *(b)* Cells from *M. smegmatis* mc^2155 and ΔPAP cultures were labelled with [^{14}-C] acetate at T1 and T2 for 1 h at 37 ºC. After organic extraction, ^{14}C-labelled lipids were separated by thin layer chromatography. Optical density standardization was performed for lipid extraction. *(c)* Quantification of the radiolabelling intensity of the TLC showed in panel *(b).* Solvent system: hexane:diethylether:acetic acid (70:30:1, v/v/v). TAG, triacylglycerol; FFA, free fatty acids; DAG, Diacylglycerol; WT, *M. smegmatis* mc^2155; AU, arbitrary units.

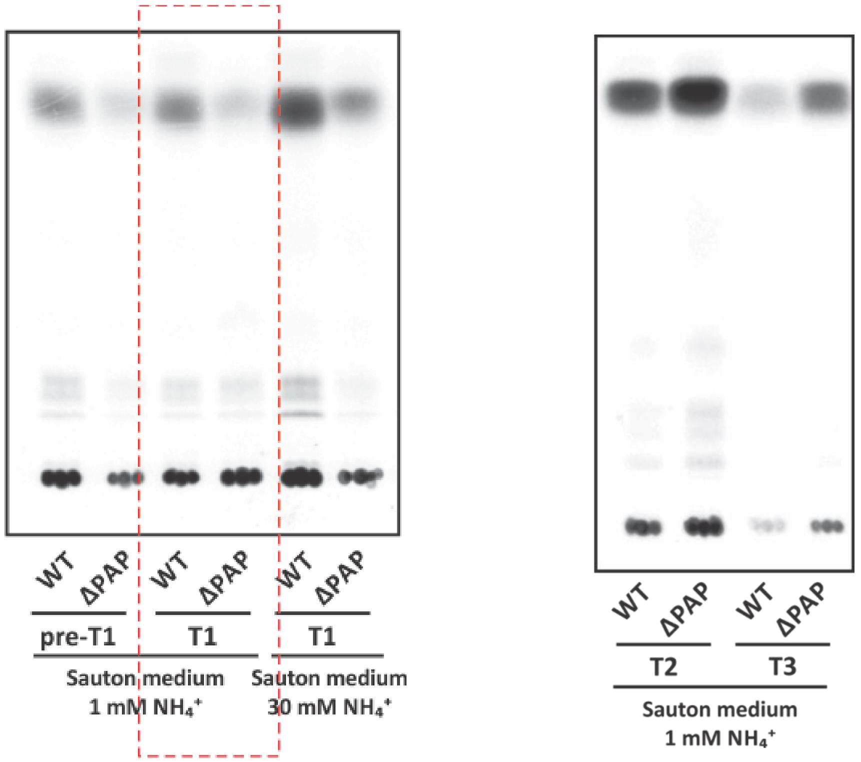

Figure S6. Full length (uncropped) image corresponding to the TLC shown in Figure 3 (b). Cells from WT and ΔPAP cultures were grown in Sauton medium supplemented with 1 mM NH_4Cl and labeled with [^{14}C]-acetate at different time points for 1 h at 37 ºC or grown in Sauton medium supplemented with 30 mM NH_4Cl and labeled with [^{14}C]-acetate at T1 for 1 h at 37 ºC. After organic extraction, ^{14}C-labelled lipids were separated by TLC. Optical density standardization was performed for lipid extraction.

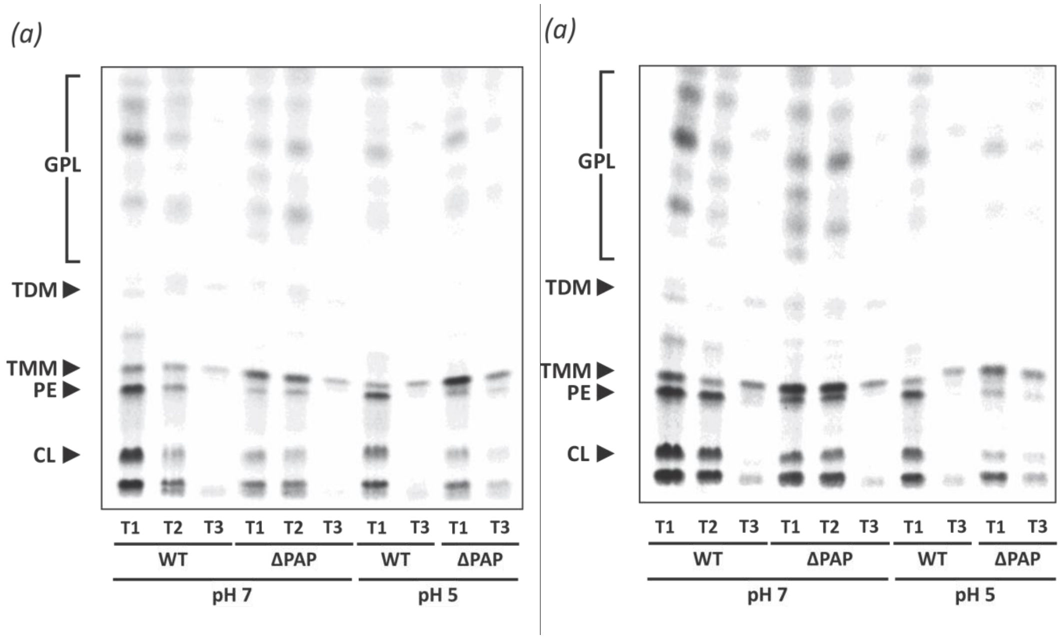

Figure S7. Multiple exposure images corresponding to Figure 5. Cells from WT and ΔPAP cultures grown in 7H9 medium (pH 7 and 5.4) were labelled with [^{14}C]-acetate at early (T1), mid-exponential (T2) and stationary (T3) phase for 1 h at 37 ºC. Cultures were normalized by OD and after organic extraction; ^{14}C-labelled lipids were separated by TLC.

References

1. Arabolaza, A., Rodriguez, E., Altabe, S., Alvarez, H. & Gramajo, H. Multiple pathways for triacylglycerol biosynthesis in *Streptomyces coelicolor*. *Appl. Environ. Microbiol.* **74**, 2573–2582 (2008).

2. Hanahan, D. Studies on transformation of *Escherichia coli* with plasmids. *J.Mol.Biol.* **166**, 557–580 (1983).

3. Comba, S., Menendez-Bravo, S., Arabolaza, A. & Gramajo, H. Identification and physiological characterization of phosphatidic acid phosphatase enzymes involved in triacylglycerol biosynthesis in *Streptomyces coelicolor*. *Microb. Cell Fact.* **12**, 9 (2013).

4. Snapper, S. B., Melton, R. E., Mustafa, S., Kieser, T. & Jacobs Jr., W. R. Isolation and characterization of efficient plasmid transformation mutants of Mycobacterium smegmatis. *Mol.Microbiol.* **4**, 1911–1919 (1990).
